# Controlling of Electrospray Deposition for Micropatterns

**DOI:** 10.3390/mi9020072

**Published:** 2018-02-06

**Authors:** Jiaxin Jiang, Gaofeng Zheng, Ping Zhu, Juan Liu, Yifang Liu, Xiang Wang, Wenwang Li, Shumin Guo

**Affiliations:** 1 Department of Instrumental and Electrical Engineering, Xiamen University, Xiamen 361005, China; jiangjx@xmu.edu.cn (J.J.); 19920151153707@stu.xmu.edu.cn (P.Z.); cecyliu@xmu.edu.cn (J.L.); yfliu@xmu.edu.cn (Y.L.); 2 Xiamen Key Laboratory of Optoelectronic Transducer Technology, Xiamen 361005, China; 3 Fujian Key Laboratory of Universities and Colleges for Transducer Technology, Xiamen 361005, China; 4 School of Mechanical and Automotive Engineering, Xiamen University of Technology, Xiamen 361024, China; wx@xmut.edu.cn (X.W.); xmlww@xmut.edu.cn (W.L.); 5 School of Mathematical Sciences, Xiamen University, Xiamen 361005, China; shumin_guo@xmu.edu.cn

**Keywords:** near-field electrospray, micropatterns, atomized particles, deposition controlling

## Abstract

Based on the electrohydrodynamic (EHD) theory, a novel method of near-field electrospray is proposed to fabricate micropatterns with micro/nano-scale particles. Compared with conventional electrospray technology, the deposition area can be decreased to print a regular pattern according to the moving trajectory of the substrate by shortening the distance between the nozzle and the collector to several millimeters in near-field electrospray. The controlling strategies in the near-field electrospray deposition process were investigated. The line width of printed pattern increased with the increase of applied voltage, deposition time, and flow rate of solution. However, it decreased with the increase of motion velocity of the substrate. By applying a suitable matching of electrospray parameters, the regular patterns with a line width under 500 μm were printed controllably on the substrate. Thereby, atomized particles from near-field electrospray were successfully deposited in specific patterns. Characters of ‘2’, ‘7’, and ‘9’ with uniform width and steady shape were patterned. This work provides an excellent way to promote the precision integrated manufacturing of electronic system.

## 1. Introduction

In recent years, the printing of micropatterns with micro/nano-particles has stimulated considerable interests of many researchers. Several techniques have been developed in the fabrication of micro/nano-particles, such as photolithography, ink jetting, plasma spraying, and pulsed laser deposition [1,2]. Compared with other techniques, electrospray is an attractive method for the fabrication of micropatterns owing to the advantages of simple equipment, low cost, flexibility, simple process, and versatile material compatibility, which lead to enormous potential applications in the fields of sensors [3], biomedicine [4], micromanufacturing [5], and flexible electronics [6].

Electrospray is a simple method for liquid atomization under the electric field force. The system can be established by supplying viscous liquid through a capillary nozzle which is maintained at high electrical potential. The diameters of atomized droplets and particles can be ranged from hundreds of micrometers down to several tens of nanometers with nearly monodisperse distribution [7]. During traditional electrospray with a large distance between the nozzle and substrate, the charge repulsion force makes the charged particles step into instable motion and increases the deposition area, which is an obstacle for micropattern deposition and system integration application of electrospray particles. 

To fabricate micropatterns, various template/molding strategies were designed to optimize the electrospray process. Munir et al. [8] and Higashi et al. [9] proposed a method to achieve micropatterns by using a corresponding mask to control the deposition process. Xie et al. [10] applied high voltage directly to the mask, which generated an electrostatic focusing effect to achieve the patterned precision deposition of electrospray particles. However, these methods with complex apparatuses are difficult to operate. At present, simple and low-cost methods are desired urgently for micropattern deposition in the preparation of integration devices in micro/nano-systems. 

As a typical technology of electrohydrodynamic (EHD) printing, electrohydrodynamic direct-write (EDW) has been widely investigated in recent years. Thanks to the short distance between nozzle and collector, a stable and straight jet is obtained for the controllable deposition of micro/nano-printed structures [11,12]. Based on the theory, the method of near-field electrospray was proposed to print micro droplets into designed patterns. Duan et al. [13] printed a simple straight structure with a line width of 35 μm by near-field electrospray and obtained a micro pentagram pattern without shape deformation successfully. Li et al. [14] used a stainless steel probe as the spinneret and reported that the electrospray process could be promoted by increasing the solution conductivity and applied voltage. Due to the charge repulsion between electrospray droplets, the precision deposition of micropatterns is still an obstacle for the application of near-field electrospray. 

In this work, a single-step process was proposed to print micropatterns using near-field electrospray without any masks. The effects of the flow rate of liquid, the velocity of substrate, the applied voltage, and the electrospray time were investigated. Various micropatterns were achieved by controlling the processing parameters and motion trajectory of substrate during the electrospray process.

## 2. Materials and Methods

**Figure 1:**
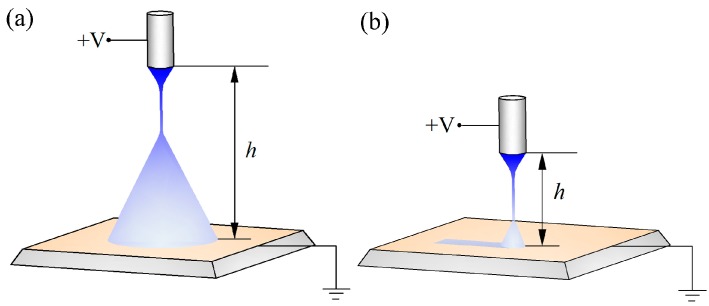
The diagram sketches of electrospray. (**a**) Conventional electrospray; (**b**) near-field electrospray.

The schematic diagram of the electrospray experimental setup was presented in Figure 1. It was normally tens of centimeters between the nozzle and the substrate in conventional electrospray process. The long spraying distance led to a large deposition area owing to the Coulombic repulsion force among charged droplets, as shown in Figure 1a, which could not be used to produce micropatterns. To suppress the self-expansion, the method of near-field electrospray, in which the spraying distance was shortened to several millimeters, was proposed to realize the regular deposition of micropatterns, as shown in Figure 1b. However, when the spraying distance was shorter than 3 mm, with the applied voltage of 1.5 kV, corona discharge would occur and the solution would not have enough time to atomize. Therefore, in this work, the spraying distance was set to be 5 mm to overcome the corona discharge and a stable spray jet was obtained. It was worth noticing that the optimal parameters varied with the spraying distance which could be adjusted by a *z*-axis motor.

As shown in Figure 1b, a high electrical field was generated between the stainless-steel nozzle with an inner diameter of 60 μm and the collector by a direct-current voltage source (DW-SA403-1ACE5, Dongwen high voltage power source Ltd., Tianjin, China). A syringe pump (Pump 11 Pico Plus Elite, Harvard Apparatus America, Holliston, MA, USA) was utilized to supply the conductive solution to the nozzle at a constant flow rate. A silicon substrate was fixed on an *x–y* motion stage (GXY1515GT4, Googoltech, Shenzhen, China) connected to the ground. The motion velocity and trajectory of the stage was controlled by a host computer to guide the pattern deposition during the electrospray process. The morphology of deposited micropattern was captured by an optical microscope (FS-70, Mitutoyo, Kanagawa, Japan), and the line width of electrospray patterns was analyzed by an image processing and analysis software (ImageJ, Version 1.51k, National Institutes of Health (NIH), Bethesda, MD, USA). The average line width and error bars in the experimental results were calculated from more than 50 data points in 10 samples.

In this work, zinc acetate dihydrate (Zn(CH_3_COO)_2_·2H_2_O) (Mocular weight = 219.51 g/mol, Sinopharm Chemical Reagent Co., Ltd., Shanghai, China) was dissolved in the solvent of glycerol to prepare the aqueous solution for the fabrication of micro-patterns. The concentration of the solution was 4 wt %, with viscosity and surface tension of 856 mPa·s and 65 mN/m. Before the experiment, the silicon substrate was ultrasonically cleaned in acetone for 20 min and then dried with air flow.

## 3. Results and Discussions

**Figure 2:**
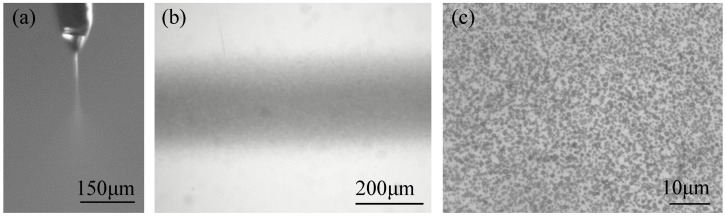
The printing process of near-field electrospray. (**a**) Ejection and motion behavior of the electrospray jet; (**b**) optical image of the deposited electrospray line; (**c**) optical image of the electrospray nanoparticles. The applied voltage, the flow rate of solution, the velocity of substrate, the distance between nozzle and collector, and the deposition time were 1.5 kV, 50 μL/h, 5 mm/s, 5 mm and 2 s, respectively.

The ejection and motion behaviors of the electrospray jet were shown in Figure 2a. The charged liquid droplets attached to the nozzle tip deformed into a conical shape under the high electrical field force. Reciprocally, the sharper curvature of the cone enhanced the electrical field. When the electrical field force increased to overcome the surface tension of the droplet, a fine jet ejected from the cone. However, due to the low solution viscosity, the jet was unstable and broke up into fine droplets. With the evaporation of solvent, the charge density on the surface of the charged jet continuously increased, resulting in the disintegration of the droplet into smaller ones before reaching the substrate. The droplets dispersed by the strong Coulombic repulsion between charges of the same polarity, and deposited into an electrospray line with the help of moving substrate, as shown in Figure 2b. The diameters of the electrospray nanoparticles were about 650~830 nm, as shown in Figure 2c.

**Figure 3:**
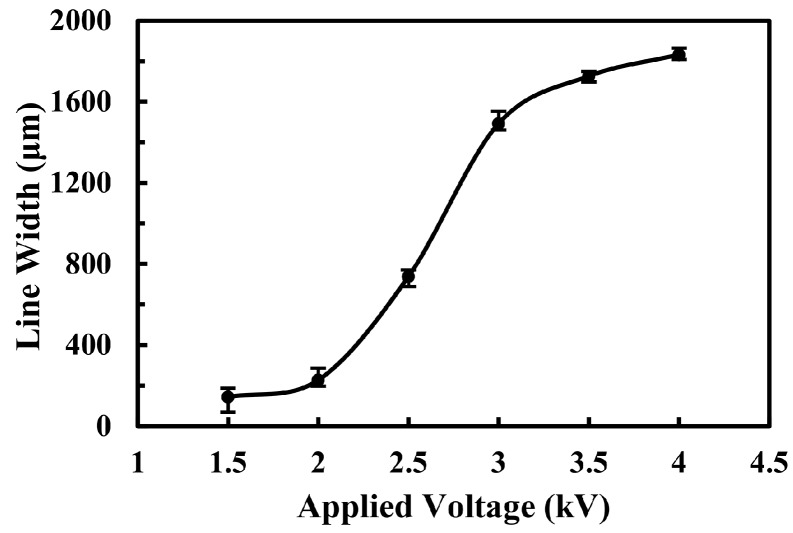
The effect of applied voltage on the electrospray line width. The flow rate of solution, the velocity of substrate, the distance between nozzle and collector and the deposition time were 50 μL/h, 5 mm/s, 5 mm and 2 s, respectively.

To reveal the controlling mechanism of near-field electrospray, the effects of processing parameters on the characteristics of printed patterns were further investigated. The applied voltage was one of the key factors affecting the ejecting behaviors of electrospray jet. The droplets would break up when the applied voltage increased to 1.2 kV, which performed as a critical state. However, at this state, the ejection of the jet was intermittent. It became stable as the applied voltage increased to 1.5 kV. With the applied voltage increased within a limited range, the electrospray process became more stable and the quantity of breaking-up droplets increased significantly. However, the nanoparticles distributed more dispersedly as the repulsive force between charges on the surface of droplets increased under a higher electrical field, which made it a barrier for the preparation of micro/nano-structures with high integration. The relationship between the line width of electrospray pattern and the applied voltage was depicted in Figure 3. It was indicated that the electrospray line width increased from 144.3 ± 75 μm to 1832.9 ± 32 μm when the applied voltage changed from 1.5 kV to 4.0 kV. When the applied voltage was higher than 4.0 kV, the high-density charges led to a strong electrical discharge that blocked the fabrication of regular electrospray nanoparticles. Therefore, lower applied voltage was more desirable to print micropatterns in the electrospray process.

**Figure 4:**
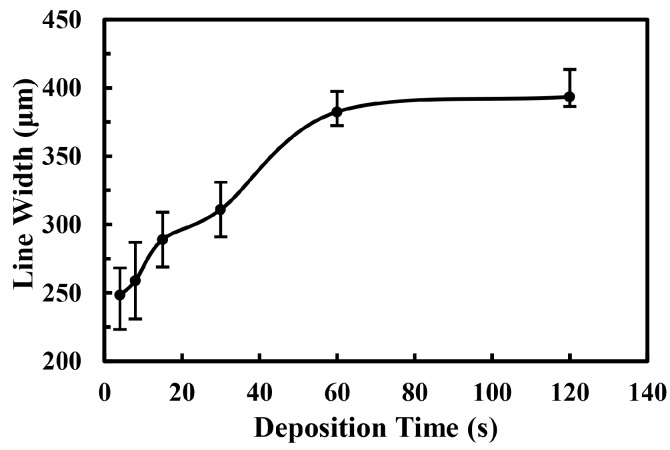
The effect of deposition time on the electrospray line width. The applied voltage, the flow rate of solution, the velocity of substrate, the distance between nozzle and collector were 1.5 kV, 50 μL/h, 5 mm/s, and 5 mm, respectively.

Then the effect of deposition time on the line width of electrospray patterns was discussed. When the deposition time increased, both the amount of charged droplets and the accumulated charges on the collector increased, so that the repulsive force became stronger, which resulted in a larger deposition area. In addition, the dispersed atomized particles tended to gather gradually, and the thickness of deposited electrospray line increased as well. However, due to the surface tension of droplets, the deposited particles merged into the bigger ones as time increased, limiting its applications for the printing of electrospray micropatterns. The relationship between line width of electrospray pattern and the deposition time was shown in Figure 4. When the deposition time increased from 4 s to 60 s, the electrospray line width increased from 248.2 ± 25 μm to 382.4 ± 15 μm in an almost linear relationship. When the deposition time was more than 60 s, the line width showed flat growth with deposition time. The reason for the small growth rate was the constant spray angle. As time increased, the thickness of the electrospray patterns raised. Fewer dispersed nanoparticles reached the edge of electrospray line and the charge repulsion became weaker. Thus, the line width varied slightly while the thickness increased largely from 62 nm to 83 nm when the deposition time increased from 60 s to 120 s. The average electrospray line width was kept below 400 μm, which could be used for the fabrication of micropatterns well under the applied voltage of 1.5 kV.

**Figure 5:**
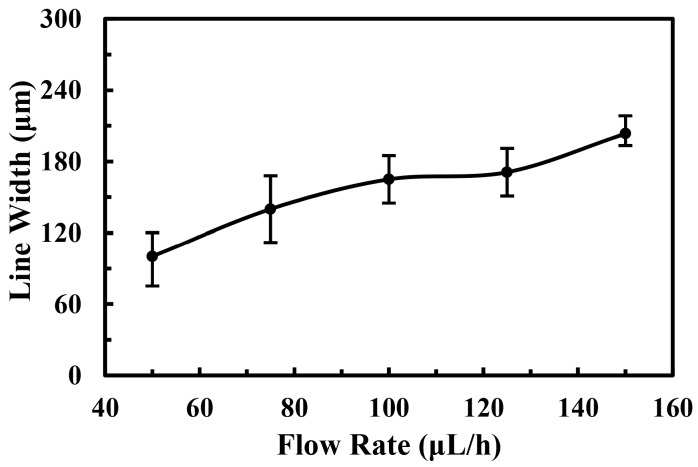
The effect of solution flow rate on the electrospray line width. The applied voltage, the velocity of substrate, the distance between nozzle and collector, and the deposition time were 1.5 kV, 5 mm/s, 5 mm, and 2 s, respectively.

The flow rate of solution was also an important factor for the electrospray process. As found in previous studies, to get a continuous and stable jet, the flow rate of solution should be kept above 25 μL/h. It was obvious that the production of atomized particles could be improved by a higher flow rate with other process conditions unchanged. However, when the flow rate increased further, the solvent could not evaporate sufficiently, thus the droplets would aggregate easily. The relationship between the line width of electrospray pattern and the flow rate of solution was shown in Figure 5. The quantities of the electrospray particles, as well as the deposition area, increased with the flow rate. Experimental results showed that the electrospray line width increased from 100.3 ± 5 μm to 203.4 ± 50 μm monotonically when the flow rate increased from 50 μL/h to 150 μL/h.

**Figure 6:**
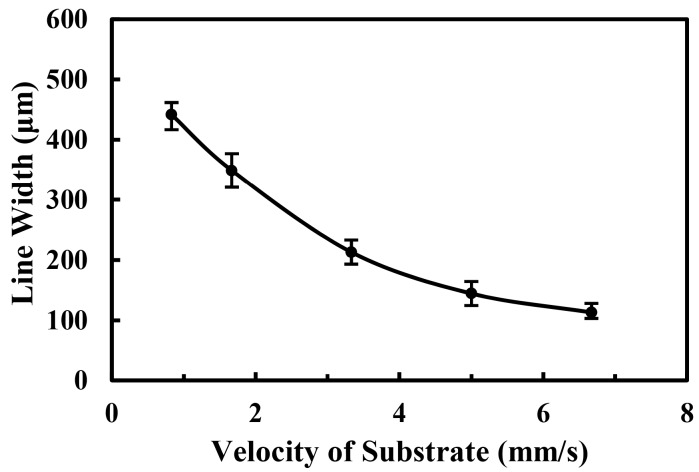
The effect of the velocity of substrate on the electrospray line width. The applied voltage, the flow rate of solution, the distance between nozzle and collector and the deposition time were 1.5 kV, 50 μL/h, 5 mm, and 2 s, respectively.

The motion velocity of the substrate was another key parameter for controlling the morphology of electrospray micropatterns during the deposition process. When the velocity of the substrate was higher than the ejecting velocity of the jet, pattern with smaller line width can be gained attribute to less deposition time. Therefore, the electrospray line width decreased with the increase of the velocity of substrate. The relationship between the line width of the electrospray pattern and the velocity of substrate was shown in Figure 6. The line width was 441.8 ± 52 μm when the velocity of substrate was 0.83 mm/s, and it decreased to 112.9 ± 9 μm when the velocity of substrate increased to 6.67 mm/s.

**Figure 7:**
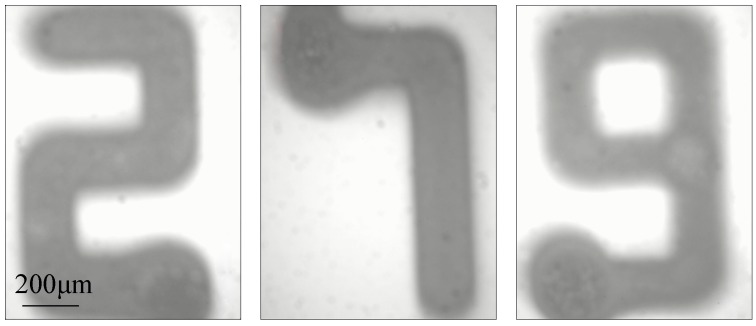
Optical images of Arabic number ‘2’, ‘7’, and ‘9’. The applied voltage, the flow rate of solution, the velocity of substrate, the distance between nozzle and collector and the deposition time were 1.5 kV, 50 μL/h, 5 mm/s, 5 mm, and 15 s, respectively.

The effects of applied voltage, deposition time, flow rate of solution, and velocity of substrate on the printing of electrospray micropatterns were discussed above. By matching these processing parameters, controllable deposition was demonstrated to obtain complex micropatterns. Through near-field electrospray, atomized particles were successfully deposited in specific patterns, which was too complex to be achieved by conventional electrospray. For instance, characters of ‘2’, ‘7’, and ‘9’ with average thickness of 38 nm were patterned by controlling the deposition trajectory, as shown in Figure 7. From the results, it could be seen that the line width of electrospray patterns was uniform, even at the corner. However, it was worth noticing that the line width slightly increased at the starting points. More particles deposited at the starting point of the micro/nano-structures due to the accumulation of liquid at the nozzle tip before the ejection of charged jet [15].

## 4. Conclusions

Near-field electrospray was introduced to improve the controlling level of deposition process for micropattern printing. For solution of low viscosity, when the electrical field force was higher than the surface tension of droplets, a fine jet would eject from the droplet attached to the nozzle and break into several tiny droplets with diameters ranging from hundreds of micrometers down to several tens of nanometers before reaching the substrate. The deposition area decreased with the distance between the nozzle and substrate, and uniform nanoparticles would be deposited into a line pattern with the help of motion stage. The controlling strategies of deposited electrospray patterns were investigated as well. As the electrospray process proceeded, the repulsive force became stronger, and the average line width increased from 248.2 μm to 382.4 μm with the increase of the deposition time. Additionally, the line width of electrospray pattern increased with the applied voltage and the flow rate of solution as well. The motion velocity of substrate was another main factor for controlling the fabrication of complex patterns. Because of the mechanical stretching force, the average line width decreased steeply from 441.8 μm to 112.9 μm when the velocity of substrate increased from 0.83 mm/s to 6.67 mm/s. Various micropatterns such as the characters of ‘2’, ‘7’, and ‘9’ were printed with average line width below 500 μm. This work was expected to promote the application of printing technologies in the fields of integration manufacturing of micro/nano system.
